# Gestational Diabetes Mellitus: Unveiling Maternal Health Dynamics from Pregnancy Through Postpartum Perspectives

**DOI:** 10.12688/openreseurope.18026.2

**Published:** 2024-11-12

**Authors:** Marina Mora-Ortiz, Lorenzo Rivas-García

**Affiliations:** 1 Lipids and Atherosclerosis Unit, Internal Medicine, Reina Sofia University Hospital, Córdoba, Andalucía, 14004, Spain; 2 GC09-Nutrigenomics and Metabolic Syndrome, Maimonides Biomedical Research Institute of Cordoba (IMIBIC), Córdoba, Andalucía, 14004, Spain; 3 Department of Medical and Surgical Sciences, Universidad de Cordoba, Córdoba, Andalucía, 14004, Spain; 4 Department of Physiology, Institute of Nutrition and Food Technology “José Mataix Verdú”, Biomedical Research Centre, Universidad de Granada, Armilla, Granada, Andalucia, 18016, Spain; 5 Sport and Health Research Centre, Universidad de Granada, Armilla, Granada, Andalucia, 18016, Spain

**Keywords:** Gestational Diabetes, Maternal Health, Foetal complications, Postpartum health, Biomarkers

## Abstract

Gestational Diabetes Mellitus (GDM) is the most frequent pregnancy-related medical issue and presents significant risks to both maternal and foetal health, requiring monitoring and management during pregnancy. The prevalence of GDM has surged globally in recent years, mirroring the rise in diabetes and obesity rates. Estimated to affect from 5% to 25% of pregnancies, GDM impacts approximately 21 million live births annually, according to the International Diabetes Federation (IDF). However, consensus on diagnostic approaches remains elusive, with varying recommendations from international organizations, which makes the comparison between research complicated. Compounding concerns are the short-term and long-term complications stemming from GDM for mothers and offspring. Maternal outcomes include heightened cardiovascular risks and a notable 70% risk of developing Type 2 Diabetes Mellitus (T2DM) within a decade postpartum. Despite this, research into the metabolic profiles associated with a previous GDM predisposing women to T2D remains limited. While genetic biomarkers have been identified, indicating the multifaceted nature of GDM involving hormonal changes, insulin resistance, and impaired insulin secretion, there remains a dearth of exploration into the enduring health implications for both mothers and their children. Furthermore, offspring born to mothers with GDM have been shown to face an increased risk of obesity and metabolic syndrome during childhood and adolescence, with studies indicating a heightened risk ranging from 20% to 50%. This comprehensive review aims to critically assess the current landscape of Gestational Diabetes Mellitus (GDM) research, focusing on its prevalence, diagnostic challenges, and health impacts on mothers and offspring. By examining state-of-the-art knowledge and identifying key knowledge gaps in the scientific literature, this review aims to highlight the multifaceted factors that have hindered a deeper understanding of GDM and its long-term consequences. Ultimately, this scholarly exploration seeks to promote further investigation into this critical area, improving health outcomes for mothers and their children.

## Plain Language Summary

Gestational Diabetes Mellitus (GDM) is a common health issue that occurs during pregnancy. It poses serious risks to both the mother and the baby, making careful monitoring and management essential. In recent years, the number of GDM cases has increased worldwide, reflecting the rise in overall diabetes and obesity rates. GDM affects a significant number of pregnancies, estimated to be between 5% to 25%. This means about 21 million babies are born to mothers with GDM every year, according to the International Diabetes Federation (IDF). There is no single agreed-upon method for diagnosing GDM, which makes research comparisons difficult. Different organizations, like the American Diabetes Association (ADA) and the International Association of Diabetes and Pregnancy Study Groups (IADPSG), have varying recommendations on how to diagnose GDM.

GDM poses different risks for the mother and the children, both, during pregnancy and after childbirth. Women with GDM face an increased risk of cardiovascular problems and have a 70% chance of developing Type 2 Diabetes (T2DM) within 10 years after giving birth. However, more research is needed to understand the specific metabolic changes that put these women at risk. On the other hand, babies born to mothers with GDM are more likely to develop obesity and metabolic issues as they grow, with a 20% to 50% increased risk.

This review highlights the need for more studies to explore the long-term health impacts of GDM on both mothers and their children. It calls for a deeper investigation into the metabolic changes caused by GDM after childbirth to better understand and manage this condition. By raising awareness and understanding of GDM, we can improve health outcomes for both mothers and their children.

## Introduction

Gestational Diabetes Mellitus (GDM) is a complex and multifactorial metabolic disorder characterized by glucose intolerance that first manifests or is recognized during pregnancy ^1, 2^. This condition poses risks to both the mother and the developing foetus, necessitating careful monitoring and management throughout the gestational period ^3, 4^. The term ‘gestational diabetes’ was first used by Carrington in 1957 ^5^, but it was not until John O’Sullivan’s publications in 1961 and 1964 that it became well-known ^6^. GDM holds significant importance for several key reasons. Firstly, it presents immediate risks to mothers both during pregnancy and in the long term ^7– 9^. Secondly, it impacts infants born to mothers with GDM, manifesting risks during pregnancy and later in life, including a heightened susceptibility to obesity and Type 2 Diabetes Mellitus (T2DM) ^4, 10– 12^. Thirdly, GDM contributes to a growing public health challenge, as its escalating prevalence parallels the global surge in obesity rates ^13^. This places strain on healthcare systems and underscores the urgent need for effective prevention strategies. Lastly, GDM can exert inter-generational effects, elevating the risk of metabolic diseases not only in offspring but also in subsequent generations ^2, 10, 14^.

Different diagnostic criteria are used depending on the organization, but all rely on specific blood sugar thresholds exceeding normal levels. The most widely accepted guidelines are provided by two different health organizations, the International Association of Diabetes and Pregnancy Study Groups (IADPSG) and the American Diabetes Association (ADA). The IADPSG recommends a universal screening approach, typically conducted between 24 and 28 weeks of gestation, utilizing a 75g oral glucose tolerance test (OGTT) ^15^. According to their criteria, GDM is diagnosed when any of the following plasma glucose values are met or exceeded: fasting plasma glucose ≥92 mg/dL, 1-hour plasma glucose ≥180 mg/dL, or 2-hour plasma glucose ≥153 mg/dL ^15^. The ADA, in its Standards of Medical Care in Diabetes, similarly recommends a two-step screening approach involving a non-fasting 50g glucose challenge test, followed by a 100g OGTT for those who screen positive. GDM is diagnosed if two or more plasma glucose values meet or exceed the following thresholds: fasting ≥95 mg/dL, 1-hour ≥180 mg/dL, 2-hour ≥155 mg/dL, and 3-hour ≥140 mg/dL ^16^. These data are summarized in Table 1.

**Table 1.** Comparison of Diagnostic Strategies for Gestational Diabetes Mellitus: One-Step vs. Two-Step Approach.

Diagnostic Strategy	Procedure	Timing	Diagnostic Criteria	Advantages	Disadvantages	References
One-Step Strategy	75-g OGTT	24–28 weeks of gestation, morning after overnight fast of at least 8 h	Fasting: ≥92 mg/ dL (5.1 mmol/L) 1 h: ≥180 mg/dL (10.0 mmol/L) 2 h: ≥153 mg/dL (8.5 mmol/L)	• Increased identification of GDM cases • Enhanced Screening for Diabetes and Prediabetes post- pregnancy • Improved Understanding of Long-Term risks	• Potential for Overdiagnosis and medicalization of pregnancies • Controversies regarding treatment impact • Concerns about unanticipated suboptimal engagement	17 18 19 20 21 22 6 23
Two-Step Strategy	**Step 1**: 50-g GLT **Step 2:** 100-g OGTT	24–28 weeks of gestation, nonfasting Fasting	If 1 h glucose level is ≥130, 135, or 140 mg/dL (7.2, 7.5, or 7.8 mmol/ L, respectively), proceed to Step 2. Fasting: ≥95 mg/ dL (5.3 mmol/L) 1 h: ≥180 mg/dL (10.0 mmol/L) 2 h: ≥155 mg/dL (8.6 mmol/L) 3 h: ≥140 mg/dL (7.8 mmol/L)	• More trial data support compared to the one-step method • Reduced consequences of overdiagnosis • Easier implementation • Reduced rates of adverse outcomes	• Increased Likelihood of GDM Diagnosis • Limited Support for One Elevated Value • Varied Diagnostic Thresholds • Controversies in Threshold Selection	24 25 26 27 28 29 30 31 32 33 34

The divergent suggestions put forth by expert groups highlight the existence of supporting data for each respective approach. The one-step approach appeared to be more likely to be cost-effective than the two-step strategy, and it also identified more cases of GDM, according to a systematic assessment of economic evaluations of GDM screening ^35^. Therefore, the chosen methodology should not only be clinically effective but also mindful of the potential stressors associated with the testing process, as this can influence the overall well-being of both the pregnant individual and the developing foetus.

**Figure 1.**
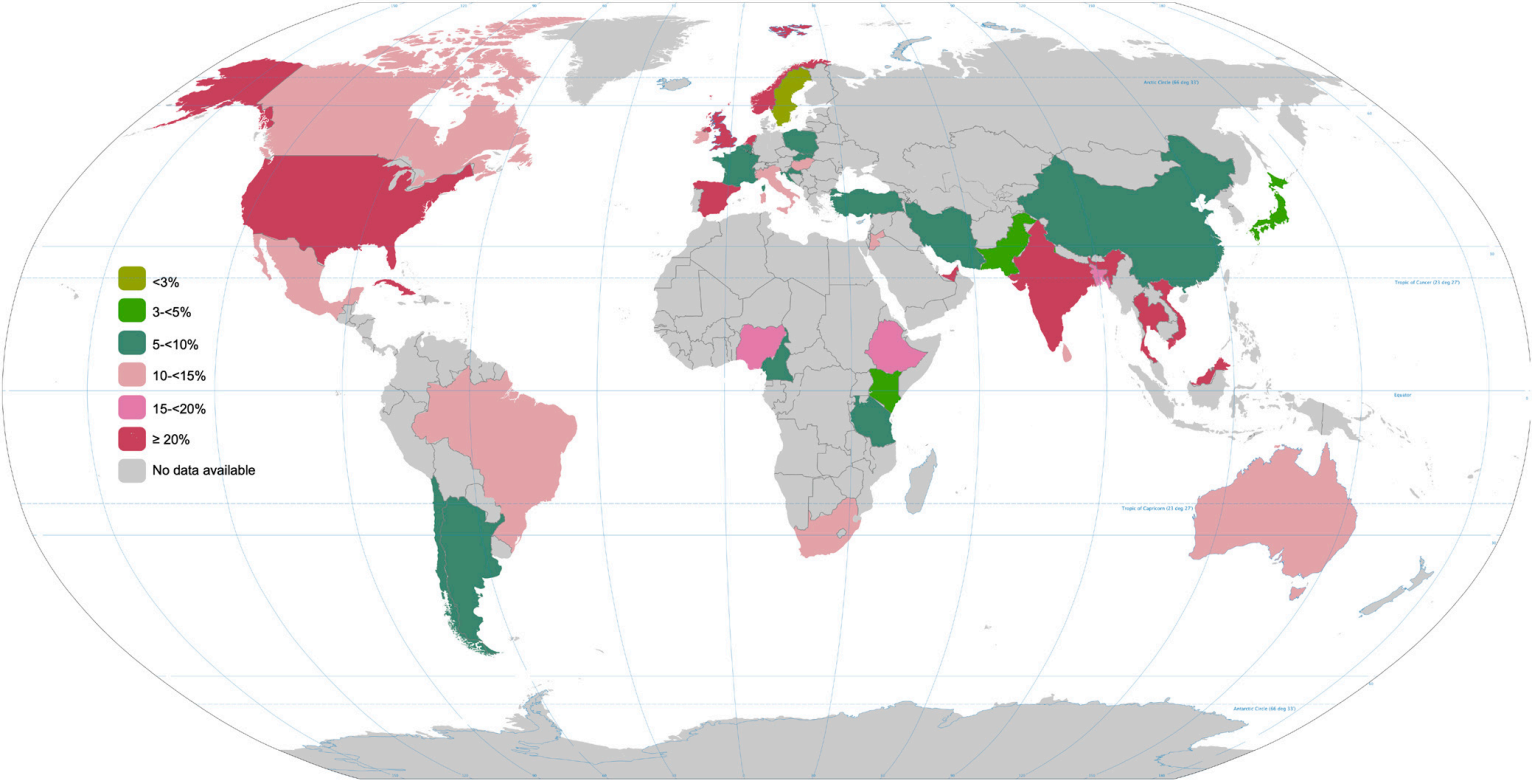
Global prevalence of GDM in 2021. This map has been generated with the data facilitated by the International Diabetes Federation (IDF) https://diabetesatlas.org/data/en/indicators/14/. It considers women between 20 and 49 years old. The data is presented in percentages.

The prevalence of GDM has witnessed a noticeable rise in recent years, paralleling the global surge in T2DM and obesity rates ^36, 37^. While estimates can vary regionally and are influenced by population demographics, lifestyle factors, and diagnostic criteria. Several epidemiological studies consistently underscore the increasing burden of GDM ^1, 38^. Thus, according to data from various countries, GDM affects a substantial proportion of pregnancies, with prevalence rates ranging from 5% to 25% ^31, 39^. This variation is influenced by different diagnostic strategies and subpopulations, making direct comparisons challenging ^39, 40^. Nowadays, the International Diabetes Federation (IDF) estimates that approximately 21 million live births are affected by GDM globally each year ^41^ ( Figure 1). Evidence shows that the global prevalence of GDM is on the rise, potentially influenced by factors like increasing maternal age, rising body mass index (BMI), and improved screening practices ^41, 42^. These issues, coupled with the recognition of GDM as a risk factor for adverse maternal and foetal outcomes, underscore the need for more targeted and effective diabetes prevention and management strategies globally ^39^. Furthermore, some authors have suggested that many cases of GDM could be in reality undiscovered cases of hyperglycaemia before pregnancy ^2^.

Here, we explore the definition and prevalence of GDM, examining the risk factors associated with its onset. Additionally, we delve into the established pathophysiology of GDM, including the molecular biomarkers identified as predictive tools for diagnosing GDM and anticipating post-pregnancy complications. Furthermore, we review current medical approaches to managing GDM. Next, we categorize post-pregnancy complications as short- and long-term issues, with particular attention to maternal health. Finally, we outline future perspectives and emphasize the most pressing knowledge gaps that require further investigation.

## A comprehensive exploration of the risk factors shaping susceptibility to GDM

GDM is influenced by a complex interplay of various risk factors, each contributing to the overall susceptibility of pregnant women to develop glucose intolerance during gestation. While the exact cause of GDM remains under investigation, several well-established risk factors contributed to its development, shedding light on the multifactorial nature of GDM. One of the most relevant risk factors is advanced maternal age, which has been consistently identified as a significant determinant for GDM ^31, 39^ ( Table 2). This is due to the physiological changes associated with ageing, including decreased insulin sensitivity, which may contribute to an increased likelihood of developing GDM ^43– 45^. For example, in a previous study women over 40 years old had a prevalence of 9.8% of GDM, while women who were under 30 years old had 4.1% ^46^.

**Table 2.** Factors Influencing Gestational Diabetes Mellitus (GDM).

Categories	Risk Factors	Description	References
**Maternal Factors**	Advanced Maternal Age	Increased risk due to ageing, and decreased insulin sensitivity.	31, 39, 43– 45
	Elevated BMI (Obesity)	Obesity-related insulin resistance, and impaired glucose metabolism.	37, 47, 48
	Previous GDM and Preeclampsia History	Higher risk for those with a history of GDM or preeclampsia.	49– 51
	Family History of Type 2 Diabetes	Genetic predisposition and familial clustering of diabetes.	10, 52– 54
	Genetic Predisposition and Type 2 Diabetes Risk Gene Variants	Presence of specific gene variants linked to increased risk.	52
	Polycystic Ovary Syndrome (PCOS)	Hormonal imbalances, hyperinsulinemia, insulin resistance.	55– 58
**Environmental and** **Lifestyle Factors**	Ethnicity	Varied risk among different ethnic groups.	59– 61
	Socio-economic Status	Lower socio-economic status linked to increased risk.	62– 64
	Dietary Habits and Physical Activity	High refined carbohydrates linked to risk; physical activity may reduce risk.	65– 71
**Emerging Factors**	Gestational Weight Gain	Excessive weight gain during pregnancy as a risk factor.	72
	Sleep Disturbances	Sleep disturbances (e.g., Sleep Apnea) associated with increased GDM risk.	73, 74

Elevated BMI is another well-documented and modifiable risk factor for GDM ^37, 47, 48^ ( Table 2). Obesity, often characterized by insulin resistance, is associated with impaired glucose metabolism during pregnancy, contributing to the development of GDM in women ^47, 48^. Particularly, a BMI over ≥ 25kg m ^-2^ has been identified as one of the most significant risk factors ^75^. Furthermore, women who have had GDM in a previous pregnancy are at increased risk for developing it again in subsequent pregnancies ^49, 50^. This risk is further heightened by the presence of preeclampsia in the first pregnancy ^51^. Similarly, a family history of type 2 diabetes in a first-degree relative (parent, sibling) significantly increases the risk of GDM ^10, 52, 53^. This risk is particularly high when the family history shows that both parents have type 2 diabetes ^54^. This score is further heightened by the presence of type 2 diabetes risk gene variants ^52^, highlighting the impact of genetic predisposition to impaired insulin action or secretion. Finally, women with Polycystic Ovary Syndrome (PCOS) have a significantly higher probability of developing GDM ^55– 57^. This may be due to the hormonal imbalances associated with PCOS, including hyperinsulinemia (elevated insulin levels) and insulin resistance. This high probability is further exacerbated by the presence of hyperandrogenism, ovulatory dysfunction, and polycystic ovarian morphology, which are key features of PCOS ^58^.

In addition to individual-related factors like maternal age or obesity, other factors highlight the broader contextual influences shaping susceptibility to GDM. For example, some studies have highlighted that ethnicity is a risk factor predisposing towards GDM. It has been reported that women of South Asian, Hispanic, African, and Middle Eastern descent are at an increased risk ^59– 61^. These ethnic disparities highlight the importance of considering diverse demographic factors in risk assessment; however, it is complicated to extract conclusive information from these disparities due to the confounding differences in socio-economic status. Indeed, socio-economic factors have been consistently associated with an increased probability of GDM. For example, studies in China ^62^ and Australia ^63^ found that lower socioeconomic status was a significant predictor of GDM. This was further supported by Cullinan (2012), who identified a strong socioeconomic gradient in GDM prevalence ^13^. However, Khan (2013) found no significant difference in GDM prevalence based on socioeconomic status in Pakistan ^64^. These findings suggest that while socioeconomic factors may play a role in GDM risk, the relationship may be influenced by other factors such as cultural and regional differences.

**Figure 2.**
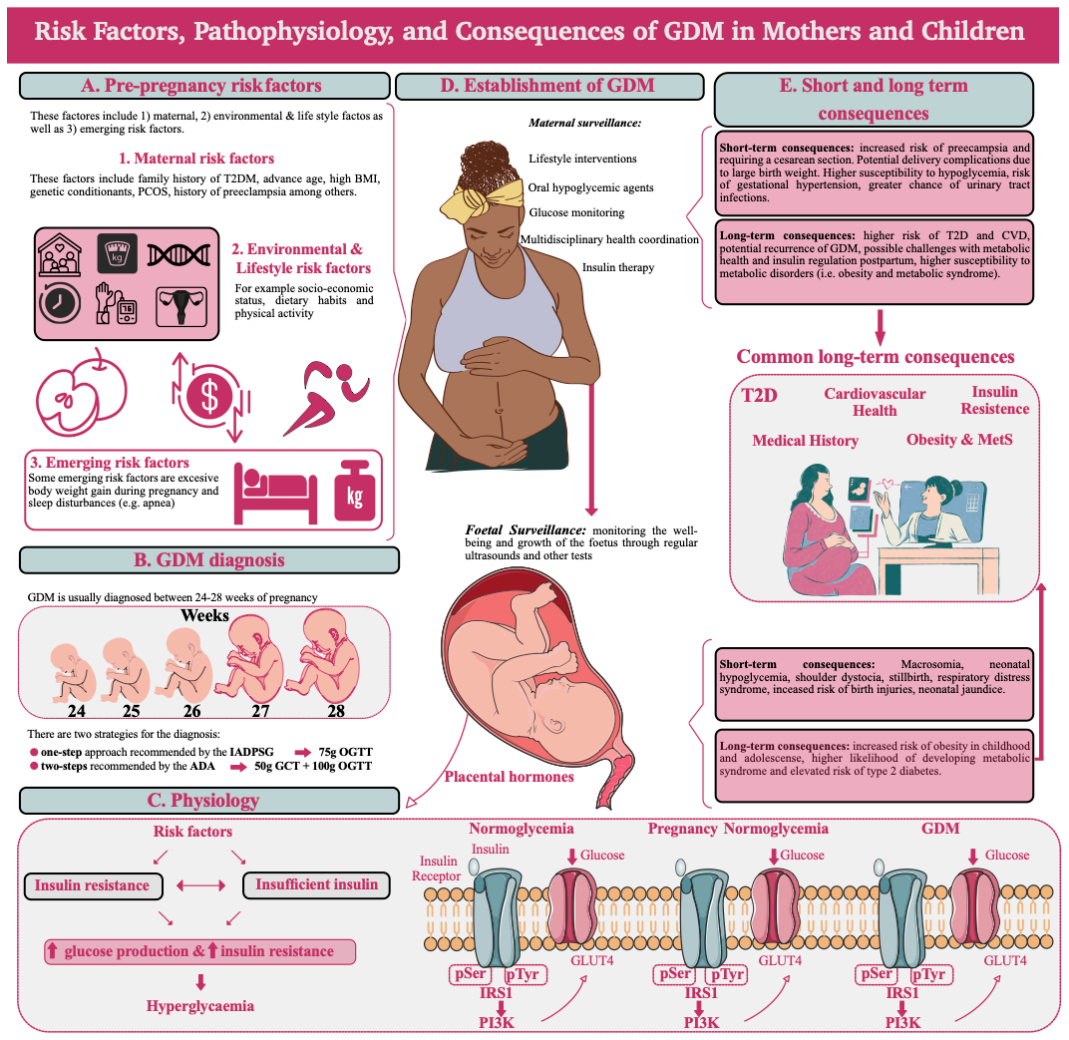
Overview of GDM risk factors, pathophysiology, and consequences for the mother and the children. **A**) There are several risk factors influencing GDM, the maternal risk factors consider for example family history of T2DM, advanced age, and high BMI among others. However, there are also lifestyle risk factors such as socio-economic status, dietary habits, and physical activity, these are interconnected. Finally, some researchers are considering emerging risk factors such as weight gain during pregnancy and sleep disturbances like apnoea. **B**) GDM is usually diagnosed between the 24 and 28 weeks of pregnancy, this is between the end of the second trimester and the beginning of the third one. The diagnosis is usually carried out following the one-step or two-step methods, recommended by the IADPSG or ADA respectively, which make comparison across studies complicated. **C**) The beginning of the GDM is linked with metabolic modulations which include insulin resistance from the maternal cells and excessive demand for B-cell activity, insulin is then inefficient in blocking endogenous glucose production and glucose uptake by the adipose tissue and skeletal muscle, producing generalized hyperglycaemia in the mother. This increased level of glucose in the mother impacts the glucose transfer through the placenta. During pregnancy in women with normal glucose tolerance, insulin signalling necessitates the tyrosine autophosphorylation of the insulin receptor within skeletal muscle. This marks the initial phase in the insulin signalling pathway, facilitating the recruitment and activation of downstream effectors like IRS1 and PI3K. Consequently, GLUT4 translocates to the plasma membrane, enhancing glucose uptake into skeletal muscle. Towards late pregnancy, the content of IRS1 in skeletal muscle diminishes compared to non-pregnant women. **D**) Once GDM is established, maternal surveillance involves actions such as lifestyle recommendations, glucose monitoring, and oral hypoglycaemic agents. In the case of the foetus, regular ultrasounds will monitor the growth. **E**) There are short- and long-term consequences for both, the mother and the foetus. Furthermore, there are common long-term consequences such as T2DM, insulin resistance, and problems of GDM incidence for both, the mother, and the female children, who will have a higher probability to develop GDM themselves. Furthermore, both sides are exposed to higher issues of obesity and metabolic syndrome.

Moreover, wealth disparities may contribute to differential access to healthcare resources and lifestyle factors, influencing the risk profile for gestational diabetes. Some of the most prevalent lifestyle factors are dietary habits and physical activity. Scientific literature has extensively shown that a high intake of refined carbohydrates and sugary drinks has been linked to an increased risk of diabetes, including GDM ^65, 66^. Conversely, a healthy diet rich in fruits, vegetables, and whole grains may offer some protective effects ^67, 68^. Similarly, sedentary lifestyles and insufficient physical activity are associated with an increased risk of GDM ^69– 71^. Finally, there are some emerging risk factors, which are gestational weight gain and sleep disturbances such as sleep apnea and other sleep disorders, which have been linked to an increased risk of GDM, possibly due to their impact on insulin regulation ^72– 74^ ( Figure 2).

Understanding the risk factors for GDM is crucial for early identification and management. Modifiable lifestyle factors like diet and exercise can play a significant role in reducing the risk. Therefore, implementing effective screening programs and interventions targeted at high-risk populations are essential strategies to ensure optimal maternal and foetal health outcomes.

## Beta-Cell Dynamics, Insulin Resistance, and Hormonal Perturbations Role in the Pathophysiology of GDM

**Figure 3.**
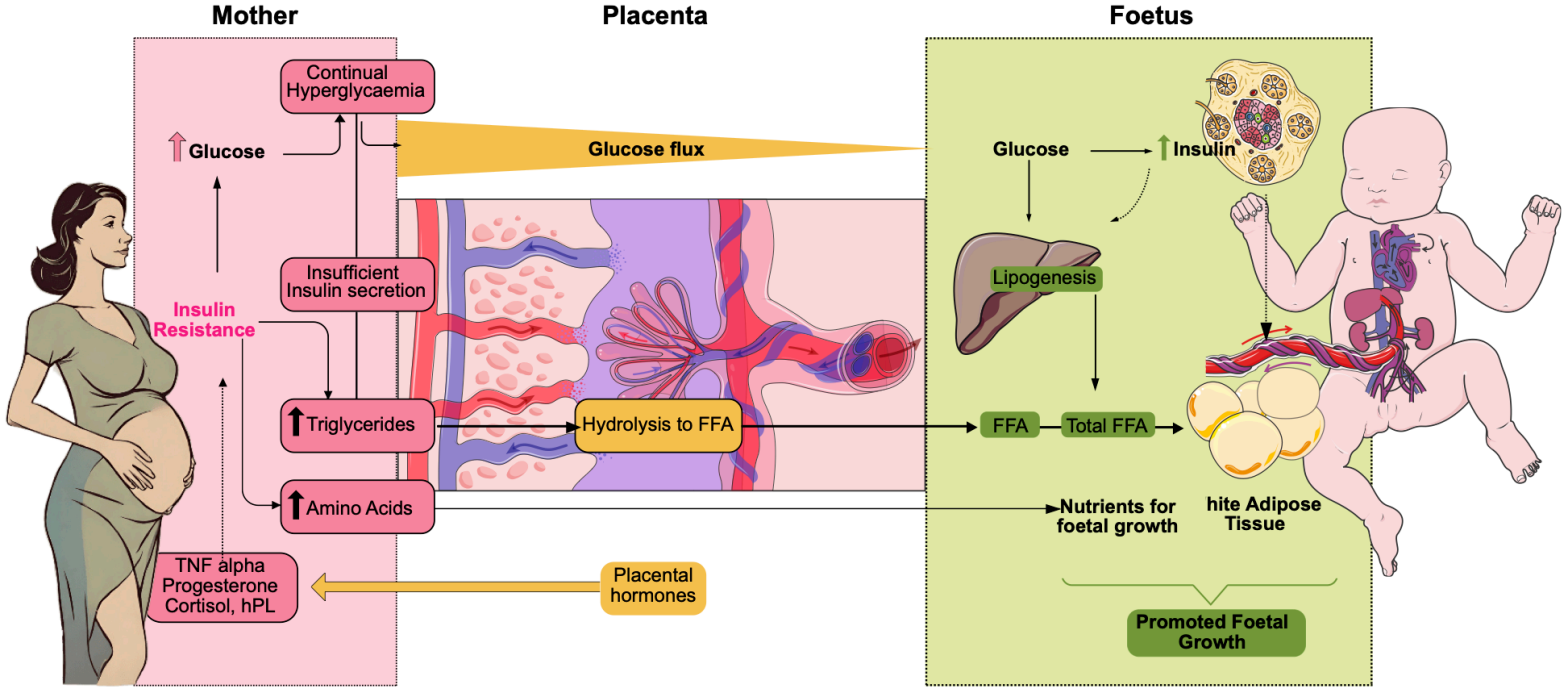
Pathophysiology of GDM. The primary macronutrient supporting foetal development maternal glucose, in women with GDM the total quantity of glucose crossing the placenta is increased. Insulin levels rise when the foetus is exposed to high blood sugar for an extended period, along with certain amino acids such as arginine and leucine. This increase in foetal insulin promotes lipogenesis in the liver and white adipose tissue production, which contributes to foetal development. This foetal growth is further enhanced in the case of women with GDM. Besides, free fatty acids (FFAs) from maternal are not abundant, but they also contribute to the total foetal FFA pool, predominantly composed of FFAs synthesized in the foetal liver from excess maternal glucose. In turn, placental hormones stimulate insulin resistance in the mother through the release of cortisol, TNF α and progesterone among others. This insulin resistance will further increase hyperglycaemia in the mother.

The pathophysiology of GDM results from pregnancy-related changes, which are primarily marked by beta-cell malfunction, insulin resistance, and hormonal imbalances caused by the placenta-foetal unit ^76^ ( Figure 3, Table 3). These changes exceed the body’s ability to preserve glucose levels, however, the exact molecular mechanisms behind this process are not completely understood ^11, 77^. According to earlier research, women who are normoglycemic but will develop GDM have a pre-existing degree of insulin resistance as a result of minor abnormalities like excessively high levels of immature insulin synthesis or unusual pulsatile insulin patterns ^78, 79^. These women’s pancreatic ß-cells can sustain normoglycemia during the first trimester by responding more strongly to insulin. However, around the end of the second and beginning of the third trimester, these adaptative mechanisms go out of balance, and it is during this time that hyperglycaemia is established that GDM is identified during ordinary medical appointments ^80^.

**Table 3.** Differences between normal physiological changes in pregnancy and changes in GDM.

System	Normal Physiological Changes in Pregnancy	Changes in Gestational Diabetes Mellitus (GDM)	References
**Endocrine System**	- Increased insulin resistance in late pregnancy due to placental hormones (e.g., human placental lactogen, cortisol, progesterone).	- Exaggerated insulin resistance. - Pancreas fails to compensate with increased insulin production, leading to hyperglycemia.	80
**Glucose Metabolism**	- Maternal glucose levels remain controlled with increased insulin secretion from the pancreas.	- High blood glucose levels (hyperglycemia) due to insufficient insulin production relative to insulin resistance.	80
**Carbohydrate Metabolism**	- Maternal body becomes more insulin resistant to ensure glucose availability for the fetus, especially in the third trimester.	- Poor glucose tolerance, higher fasting and postprandial glucose levels. - Higher risk of macrosomia (large baby) due to excessive glucose.	81
**Pancreas Function**	- Increased beta-cell hypertrophy and hyperplasia, resulting in higher insulin secretion to overcome insulin resistance.	- Pancreatic beta cells do not produce enough insulin to overcome the heightened insulin resistance, resulting in hyperglycemia.	79, 80
**Weight Gain**	- Normal weight gain of about 11.5–16 kg (25–35 pounds) based on pre-pregnancy BMI.	- Excessive maternal weight gain is common in GDM due to poor glucose control. - Increased risk of fetal macrosomia (larger baby).	72, 74
**Fetal Development**	- Normal fetal growth supported by placental glucose transfer.	- Higher glucose transfer to the fetus leads to excessive growth (macrosomia). - Risk of neonatal hypoglycemia after birth due to insulin overproduction.	82– 84

The primary macronutrient supporting foetal development is maternal glucose; however, in women with GDM the total quantity of glucose crossing the placenta is increased. Insulin levels rise when the foetus is exposed to high blood sugar for an extended period, along with certain amino acids such as arginine and leucine. This increase in foetal insulin promotes lipogenesis in the liver and white adipose tissue production, which contributes to foetal development. This foetal growth is further enhanced in the case of women with GDM. Besides, maternal free fatty acids (FFAs) are not abundant, but they also contribute to the total foetal FFA pool, predominantly composed of FFAs synthesized in the foetal liver from excess maternal glucose. In turn, placental hormones stimulate insulin resistance in the mother through the release of cortisol, TNF α and progesterone among others. This insulin resistance will further increase hyperglycaemia in the mother. By the end of gestation, the mother needs to increase glucose production by approximately 30%, primarily through hepatic gluconeogenesis. This increase is essential to meet the elevated fasting energy demands of pregnancy, ensuring a continuous supply of glucose to both maternal tissues and the growing fetus ^81^. Despite this increase in glucose production, maternal plasma glucose concentrations are typically lower, likely due to the expanded plasma volume and the increased glucose utilisation by the feto-placental unit during late gestation. Even in healthy pregnancies, there is a reduction in insulin sensitivity of approximately 50% by the end of gestation. In women without GDM, this reduced insulin sensitivity is compensated by a 2- to 3-fold increase in insulin secretion. As a result, euglycaemia is maintained ^2^.

Conversely, the pathophysiology of GDM is characterised by heightened insulin resistance and impaired β-cell function. Indeed, some studies suggest that these β-cell defects are present prior to conception but only become apparent due to the metabolic stress imposed by pregnancy ^79, 80^. In normal pregnancies, even with a natural and non-pathological increase in glucose production of about 30% to meet energy demands, endogenous glucose production is almost completely suppressed in women who had normal blood sugar levels before conception when subjected to an insulin infusion in a controlled setting known as a hyperinsulinaemic-euglycaemic clamp. This suppression is critical, as it indicates that, under normal circumstances, the body can effectively regulate glucose production in response to increased insulin levels.

In contrast, women who develop GDM exhibit significantly less suppression of endogenous glucose production—around 80-85%—compared to the nearly complete suppression (close to 100%) seen in women with normal glucose tolerance. This reduced suppression contributes to postprandial hyperglycaemia in women with GDM ^79^.

In the case of healthy pregnancies, the insulin receptor that is located on the cell surface enables the body to absorb glucose by triggering a series of events that eventually lead to the rearrangement of the glucose transporter type 4 (GLUT4), which in turn enables the body to absorb glucose. As a general rule, pregnant women have a less effective mechanism than non-pregnant ones, but women with GDM have an even less effective mechanism; as a result, more glucose stays in the blood and is not taken up by the cells ^85^. Furthermore, pregnant women have a smaller amount of the insulin receptor substrate 1 (IRS1) than non-pregnant women. IRS1 is likewise implicated in glucose uptake. In addition, GDM causes a 25% reduction in the total amount of glucose that can be potentially absorbed by the cells due to a decrease in the autophosphorylation of IRß over healthy pregnancies ^86^.

Formerly, hormones connected to the placenta have been credited for the insulin resistance issues that occur in pregnant mothers. Progesterone, cortisol, pregnancy-associated plasma protein-A (PAPP-A), and human placental lactogen (hPL), are essential for the growth of the foetus; however, in certain cases, they can simultaneously cause peripheral tissues of mothers to become insulin resistant ^12, 87, 88^. These aforementioned hormones were linked, respectively, to a rise in maternal food intake, a post-binding impairment in insulin action, and the enlargement of maternal beta cells. They are also linked to the start of GDM and control islet alterations throughout gestation ^12, 87, 88^. Insulin resistance, which mostly affects skeletal muscle and adipose tissue, reduces the body’s ability to absorb glucose, which causes the mother’s blood glucose levels to rise. Although the placenta is properly provided with transporter compounds that aid in the absorption of amino acids, lipids, and glucose, it is not made to stop an overabundance of glucose, which is what happens in the case of GDM ^89^. When the differential glucose concentration between the maternal and foetal circulation reaches 25mmll ^-1^, transplacental glucose transport hits the sensitive saturation point ^90^. Therefore, the delivery of glucose is unaffected in the event of GDM; thus, the mother’s glucose concentration is the primary factor controlling the amount of glucose that reaches the foetus ^89^. When there is GDM and high glucose arrival, the foetus also experiences hyperinsulinemia, allowing the foetus’s cells to enter glucose ^89^. Furthermore, the mother’s elevated glucose level is partially caused by the 30% rise in maternal glucose that results from the hepatic glucose release ^91^. Therefore, the admission of glucose across the placenta will be facilitated by both, maternal and foetal hyperinsulinemia. On the other hand, effective transfer is not necessary for fatty acids since the foetus may synthesise non-essential fatty acids on its own by using glucose as a precursor. The placental transfer system does, in fact, allow only around 3% of the mother’s fatty acids to reach the developing foetus; this is far less efficient than for glucose ^92, 93^.

Although placental-derived hormones have historically been associated with the occurrence of maternal insulin resistance, the exact processes by which these compounds cause insulin resistance are still unclear. Some authors have proposed that the typical reproductive hormones could not be the main drivers of the change in insulin susceptibility occurring during GDM, as Tumour Necrosis Factor-alpha (TNF-α), a cytokine implicated in immune regulation and inflammation produced by the placenta, is a significant marker of insulin resistance in advanced pregnancy ^94^. Kirwan and colleagues (2002) investigated the relationship between alterations in the sensitivity to insulin during pregnancy and modulations in TNF-α, placental hormones, leptin, and cortisol, with a small cohort of 15 women (5 with GDM and 10 with normal glucose tolerance). Interestingly, among all the hormonal shifts evaluated, the only significant predictor of the change in insulin sensitivity was the modulation of TNF-α from pre-gravid to advanced pregnancy (r = -0.60, P < 0.02). In contrast, cortisol and placental reproductive hormones did not significantly correlate with late-pregnancy insulin sensitivity ^94^. This is corroborated by the discovery that GDM promotes placental genes linked to inflammatory and chronic stress pathways, which are linked to the mother’s insulin resistance ^95^. Additionally, by increasing insulin production, the compensatory mechanism carried out by beta cells within the pancreatic islets aims to offset maternal insulin resistance ^96– 99^. Although this adaptive response operates well at first, it puts a lot of stress on beta cells ^96, 97^. Pregnancy can increase the body’s need for insulin beyond what beta cells can produce, which can result in insufficient insulin production and the subsequent development of GDM ^96– 99^. The complex interactions between metabolic, hormonal, and placental variables highlight the complex pathophysiology of GDM and highlight the need for thorough research into its molecular underpinnings to improve the disease’s management and prevention.

## Short-term and long-term complications derived from GDM in the mothers and children

Issues related to gestational diabetes mellitus have noteworthy consequences for both the expectant mother and the growing foetus during the gestational period and postpartum ^82, 100, 101^. Moreover, GDM can lead to short-term pregnancy complications, including high blood pressure, the need for a cesarean section and preeclampsia. In the long term, it may recur in future pregnancies and raises the mother's likelihood of developing T2DM later in life ^102^.

Preeclampsia and an increased risk of caesarean section are the most significant maternal problems ^103^. For example, Catalano and colleagues employed the HAPO prospective observational study, which included 25,000 pregnant women from 10 different countries, to examine the connection between preeclampsia and GDM. The volunteers of this study followed a 75-g oral glucose tolerance test (OGTT) ranging from 24 to 32 weeks, and GDM was identified based on the IADPSG criteria. Finally, the scientists concluded that GDM patients had a noticeably higher incidence of preeclampsia ^104– 106^.

However, it remains debated whether GDM and preeclampsia are independently related or share common risk factors, particularly obesity. Additionally, concerns about macrosomia, foetal distress, and other GDM-related complications may contribute to the increased risk of caesarean sections ^83, 84^. On the other hand, neonatal hypoglycaemia, macrosomia, shoulder dystocia, respiratory distress syndrome and stillbirth are typically associated with foetal and neonatal problems (RDS) ^82^. Macrosomia, a common problem in GDM-affected pregnancies marked by abnormal foetal growth, is caused by the mother's elevated insulin resistance, which can result in a larger amount of blood glucose entering the foetal circulation across the placenta ^84^. Increased insulin synthesis in the foetus can be caused by elevated maternal glucose levels, which could speed up the child’s growth ^14, 83^. As such, macrosomic babies are more likely to experience birth trauma and require caesarean sections ^84, 107, 108^. Ensuring rigorous glycaemic management, specifically aiming for a mean blood glucose level of 5.3 mmol/l, can considerably lower the incidence of macrosomia ^109^. In addition, strict metabolic control — which involves a diet restricted in fat and oligosaccharides, and insulin treatment when needed—, can reduce neonatal morbidity in GDM ^110^. Indeed, babies delivered to moms with GDM may have hypoglycaemia, which is concerning since it causes an abrupt decrease in blood glucose levels after delivery ^77, 111^. Higher adipose mass and/or larger newborns as well as those with greater cord serum C-peptide levels carry a higher risk ^112^. In these cases, effective care of newborns requires regular glucose monitoring and early breastfeeding ^111, 113^. Furthermore, babies born to GDM moms may have a higher chance of developing respiratory distress syndrome (RDS), which is a disorder marked by a pulmonary surfactant shortage ^114^. Therefore, macrosomia, altered surfactant production, and prematurity are some of the multifactorial variables that contribute to the link between RDS and GDM ^114^.

GDM may raise the risk of long-term repercussions for the mother and the child, such as an increased risk of T2DM, metabolic syndrome, and obesity, in addition to pregnancy-related problems ^10^. Furthermore, long-term consequences for the moms also include a higher chance of cardiovascular health issues and a possible chance of GDM recurrence in subsequent pregnancies ^8, 115^. Thus, women who suffered from GDM previously have a considerably greater probability of developing T2DM later in life ^115^. According to longitudinal studies such as the Diabetes Prevention Program Outcomes Study, up to 70% of mothers with a previous diagnosis of GDM will have T2DM within ten years of giving birth ^116^. Interestingly, research suggests a beneficial relationship between breastfeeding and glycemic control in postpartum women with gestational diabetes mellitus (GDM). Exclusive breastfeeding is associated with reduced fasting glucose levels compared to non-exclusive breastfeeding in women with a history of GDM ^117, 118^. Higher breastfeeding intensity correlates with improved fasting glucose, lower insulin levels, and reduced prevalence of diabetes or prediabetes at 6–9 weeks postpartum ^119^. In addition, long-term benefits include a decreased risk of developing type 2 diabetes, with breastfeeding for ≥3 months delaying its onset by up to 10 years compared to <3 months ^120, 121^. Despite these advantages, women with GDM are less likely to breastfeed or do so for shorter durations than women without GDM ^120^. These findings underscore the importance of promoting breastfeeding as a potential intervention for improving long-term health outcomes in women with GDM. Encouraging and supporting breastfeeding not only offers immediate glycemic benefits but may also play a crucial role in reducing the future risk of type 2 diabetes. By fostering a supportive postpartum environment and addressing barriers to breastfeeding, healthcare providers can help women with GDM maximize these significant health advantages for both themselves and their children.

Similarly, an elevated risk of cardiovascular disease (CVD) in subsequent years has been linked to GDM ^122, 123^. Adverse cardiovascular risk profiles, such as dyslipidaemia, endothelial dysfunction, and hypertension are present in women with a history of GDM ^124, 125^. In addition, previous research, such as the Nurses' Health Study II, indicates a significant rise in the probability of reoccurring GDM, with likelihood ratios ranging from 2 to 10 ^126^. This return highlights the ongoing impact of metabolic dysfunction and highlights the need for close observation and prompt management in future pregnancies. The possible intergenerational effects of recurrent GDM are another long-term effect; this is still a poorly studied field. Furthermore, women who have experience GDM could be more exposed to postpartum stress ^127^. The postpartum period is commonly associated with elevated stress levels, often driven by physical recovery, sleep deprivation, and the demands of infant care ^128^. For women with a history of GDM, these stressors are compounded by ongoing health monitoring, which often includes dietary restrictions, glucose testing, and follow-up assessments for diabetes. While there is growing interest in understanding the psychological impacts of GDM, current research on postpartum stress remains limited and inconclusive. The existing findings are mixed, likely influenced by individual factors such as coping strategies, social support, and the severity of GDM complications, highlighting the need for more comprehensive research in this area. For example, a study carried out in China reported that nearly half of rural women with a history of GDM had increased stress compared to those without a previous GDM ^129^. Similarly, a Danish study showed that women experienced insufficient access to healthcare provides to help to cope with all the requirements following the GDM and the childcare ^130^. In the same way, a study carried out in USA detailed that fear of receiving a T2DM diagnosis was a key barrier in the mental health of women who have experienced GDM ^127^. Conversely, another study showed not differences in anxiety scores between women with GDM and the control group during the postpartum period ^131^. Stress and anxiety can lead to chronic stress, which is a well-known risk factor for both physical and mental health complications ^132^; therefore, it will be necessary to conclude the extent to which women with GDM are particularly vulnerable to these issues and how tailored interventions can mitigate their long-term health risks.

On the other hand, long-term problems in children include a greater incidence of T2DM, obesity and metabolic syndrome ^133– 135^. Children and adolescents born to moms with GDM have a higher chance of growing up obese and having metabolic syndrome ^134, 135^. Studies using a longitudinal design, such as the “Hyperglycaemia and Adverse Pregnancy Outcomes (HAPO)” cohort, show a correlation between maternal hyperglycaemia during pregnancy and a higher risk of metabolic disorders and juvenile obesity ^25, 31^. Similarly, Children who experienced their mother's hyperglycaemia during pregnancy are more likely to grow up to have T2DM. For instance, long-lasting research, like the Helsinki Birth Cohort Study, has demonstrated that adult levels of insulin resistance and intolerance to glucose are higher in those who were exposed to GDM during pregnancy ^136^. This highlights the impact of hyperglycaemia in utero on the offspring. It is noteworthy that there remains a gap in research, and insufficient exploration of the long-term complications calls for a more extensive investigation into the enduring health implications for both mothers and their offspring, particularly in this current scenario of childhood obesity.

## Biomarker signatures in GDM

Previous studies have identified genetic biomarkers associated with GDM, illustrating its multifactorial nature involving hormonal changes, insulin resistance, and inadequate insulin secretion ^137– 139^. For instance, Shaat *et al.* (2007) reported an association between genetic variants and GDM risk, particularly a variant in the transcription factor 7-like 2 (TCF7L2) gene ^140^. MicroRNA-375 levels and single nucleotide polymorphisms (SNPs) in microRNA-375 have also been linked to GDM ^141– 144^. Beyond genetics, biomarkers like dietary patterns and hormonal levels have been investigated in relation to GDM. Dietary factors, including high advanced glycation end product (AGE) consumption, have shown associations with GDM risk ^145– 148^. Additionally, hormonal biomarkers like prolactin, progesterone, and thyroid-stimulating hormones have demonstrated potential for early GDM detection ^149^.

Recent studies have associated the presence of specific metabolites with the progression and incidence of GDM. These investigations have primarily utilized metabolomics-based techniques, chromatography (such as HPLC-MS), and immunolabeling methods (particularly ELISA). The main characteristics are summarized in Table 4.

**Table 4.** Metabolites that could conditionate GDM.

Metabolite(s)	Location	Determination technique (s)	Sample	Main results	Reference
HbA1c	New York City, USA	Chromatography	Blood	Preconception levels of HbA1c Levels could predict the risk of GDM in adolescents and young adults	150
The ratio of triglycerides to phosphoglycerides (TG_by_PG)	Norfolk, UK	Metabolomic	Blood	TG_by_PG is causally associated with an increased risk of GDM	151
-Methyltetrahydrofolate (5-MTHF) -Plasma homocysteine (HCY) -Unmetabolised folic acid (UMFA) -5, 10-methylene-tetrahydrofolate (5,10-CH2-THF) -5- formyltetrahydrofolate (5-CHO-THF)	Beijing, China	Immunoassay	Blood	Elevated levels of UMFA and HCY during early pregnancy, along with elevated red blood cells 5-MTHF and 5,10-CH2-THF and plasma 5-MTHF during mid- pregnancy, are associated with GDM	152
microRNA-125b	Chennai, India	Quantitative Real- time PCR	Blood	microRNA-125b conditionated the progression of GDM. It was downregulated in GMD	153
Mannose	Finland	Metabolomic	Serum	High levels of mannose were found to be causally associated with increased risks of GDM	154
Folate 5-MTHF	Beijing, China	Chromatography	Plasma and red blood cells	Folates and metabolites were diversely associated with GDM development	155
Up to 32, mainly Allantoic acid	Shanghai, China	Metabolomic	Serum	32 metabolites, which were clustered into three distinct patterns, were associated with GDM throughout pregnancy	156
Pentose metabolites	South Korea	Chromatography	Orine	Urinary pentose metabolites were identified as biomarkers of particulate matter 2.5 which is related to GDM	157
Polycyclic aromatic hydrocarbons (PHAs)	Region of Zunyi, China	Chromatography	Orine	PHAs were associated with an increased risk of GDM and gestational hypertension	158
-Ursodeoxycholic acid - Docosahexaenoic acid -8,11,14-eicosatrienoic acid	Guangzhou, China	Metabolomic	Umbilical cord	These metabolites was associated with well-controlled GDM	159

Metabolomics is a powerful high-throughput technique that enables the study of the complete set of metabolites within biological systems. One of its primary advantages is its broad specificity, allowing for the simultaneous detection and analysis of a wide range of small molecules. This feature is especially valuable for comprehensive studies, such as biomarker discovery or assessing metabolic responses to diseases or environmental changes. Furthermore, metabolomics offers excellent sensitivity, enabling researchers to detect subtle variations in metabolic profiles that may indicate disease progression or other physiological processes. Using metabolomics, distinct metabolic differences have been identified that may influence the incidence and progression of GDM. For instance, elevated levels of hemoglobin A1c (HbA1c) have been linked to an increased incidence of GDM in young individuals and adolescents, as well as adverse birth outcomes ^150^. HbA1c serves as an indicator of average blood glucose levels over time. Additionally, variations have been reported in other metabolites, such as lipids (including triglycerides and phosphoglycerides), specific oligosaccharides like mannose, and vitamins such as folate.

Some authors proposed that in women with GDM, lipid metabolism is notably affected, which can lead to impaired lipid metabolism and increased triglyceride levels compared to non-diabetic pregnant women. These alterations influence cellular membrane structure and function and affect overall lipid metabolism. Elevated triglyceride levels in GDM are also associated with a higher risk of cardiovascular disease and metabolic complications. Moreover, GDM induces changes in carbohydrate metabolism due to insulin resistance, potentially altering levels of certain monosaccharides, such as mannose. In the context of GDM, folate levels may be impacted by increased metabolic demands and changes in the absorption and utilization of this vitamin. Insulin resistance can also disrupt folate metabolism, while mannose is implicated in inflammatory processes and the body's response to metabolic stress. Increased levels of metabolites associated with inflammatory processes have also been observed ^157, 160^. For instance, women with GDM exhibited higher levels of IL-10, TNF-α, IL-6, lipopolysaccharide (LPS), and TLR4 compared to non-diabetic women, as measured by ELISA ^160^. These same metabolites were also elevated in GDM umbilical cord samples, indicating that oxidative stress and inflammation may mediate the release of pro-inflammatory cytokines associated with diabetes ^161^.

Additional studies in umbilical cord samples have revealed that metabolic differences between diabetic and non-diabetic women may be linked to pathways involving linoleic acid (LA) and alpha-linolenic acid (ALA) ^159^. These essential fatty acids play crucial roles in diabetes due to their involvement in various metabolic pathways and their influence on inflammation, lipid metabolism, insulin resistance, glucose homeostasis, and insulin signaling ^162^.

Furthermore, insulin resistance and glucose intolerance seen in GDM seem linked with adipose tissue dysfunction, which is characterized by altered adipokine production, poor lipid metabolism, and elevated inflammation ^7^. Additionally, research has shown certain biomarkers linked to GDM, for example, women with GDM have modified levels of leptin, an adipokine involved in regulating appetite, which may indicate an imbalance in the function of adipose tissue ^94^. The role of leptin in GDM is complex ^163^, and scientific literature is not clear yet about its modulation in the context of this disease. By stimulating signals produced from the hypothalamus, leptin plays a significant role in controlling energy expenditure and food intake. Previous studies showed that in cases of GDM without hypertension, the levels of leptin are lower than in regular conditions. However, when GDM occurs together with obesity, the overall levels of leptin increase, which has been mainly attributed to fat tissue production ^164– 166^. In addition, when the person has high levels of free fatty acids in blood, the leptin production is further exacerbated, which was attributed to the difficulty of the adipose tissue to use energy properly ^165, 166^. Furthermore, in patients with insulin resistance the levels of leptin have been shown to rise up regardless of the body fat of the patient ^165, 166^. Women who develop GDM early in their pregnancy might have higher levels of leptin compared to those who develop it later, which is attributed to inflammation and oxidative stress as a result of the imbalance between free radicals and antioxidants ^165, 166^. In addition, a study that looked at nearly 1,700 patients found that the levels of leptin in the umbilical cord are higher in pregnancies with GDM ^167^, suggesting that the baby is also producing more leptin ^167^. This same study also found that there are more soluble leptin receptors in the placenta of women with GDM, which are proteins that can bind to leptin and influence its effects. Although leptin is known to be involved in GDM, researchers have not yet fully grasped the ways in which it influences the condition.

In addition to leptin modulation, reduced levels of adiponectin, another adipokine with insulin-sensitizing effects, have also been observed in GDM, indicating a potential involvement in the development of insulin resistance ^124^. Moreover, the adipose tissue of women with GDM has been shown to have pro-inflammatory markers such as interleukin-6 (IL-6) and TNF-α, providing more evidence that adipose tissue inflammation plays a role in the pathophysiology of GDM ^168, 169^. These biomarkers offer information on the underlying biological processes of GDM as well as prospective targets for additional research and treatment treatments. However, the intricacy of GDM—which is influenced by both genetics and environment—highlights the need to research the interaction between genetic susceptibility and modifiable risk factors ^146^. Knowledge of these indicators and how they interact with genetic and environmental variables can help control GDM and understand the molecular alterations seen after pregnancy ends by assisting in early identification, prevention, and treatment. There is currently little information in the literature on these interactions, and further study is required to fully comprehend the function of genetic biomarkers in GDM both during and after pregnancy.

## Current approaches to GDM

GDM management considers i) lifestyle interventions, ii) medical therapies, and iii) vigilant monitoring throughout pregnancy to optimize maternal and foetal outcomes. It is worth mentioning that lifestyle modifications, including dietary adjustments and increased physical activity, play a pivotal role in glycaemic control and reducing the risk of complications. On the one hand, prospective and interventional studies have highlighted the need to balance carbohydrate intake, emphasizing whole grains, fruits, vegetables, and lean proteins while limiting sugary and processed foods in GDM ^71, 170^. For example, the DiGest study, a randomized controlled trial, assessed the impact of a reduced-calorie diet on pregnant women with GDM, underscoring the importance of dietary interventions in managing this condition ^170^. Despite the known association between pregnancy complications such as GDM and an elevated risk of obesity in offspring, as well as the widely recognized significance of dietary intervention, primarily gleaned from general studies on obesity and T2DM, the scientific literature reveals a paucity of interventional studies addressing this issue. Therefore, it is crucial to emphasize the need for additional studies to comprehensively understand this issue and determine the most effective strategies to address it.

On the other hand, regular physical activity, tailored to individual capabilities, promotes glucose utilization and insulin sensitivity, through the activation of AMP-activated protein kinase (AMPK), and is consequently recommended for women at risk of GDM ^171– 173^. However, the effectiveness of this approach is still under debate, possibly due to the scarcity of studies on the topic. For example, the FitFor2 study, a randomized controlled trial carried out in the Netherlands with 121 women, determined that the exercise programme followed by the volunteer women twice a week had no effects on blood glucose, insulin sensitivity, or birthweight ^174^. Similarly, another study involving 32 women found no significant effects from walking, although it acknowledged that the accumulation of short walks after meals was comparable to continuous walking for the same duration ^175^. Altogether, this suggests that there is a lack of original studies examining the effectiveness of physical activity in controlling GDM. It seems necessary to advocate for further research in this field to better understand the role and effectiveness of physical activity in GDM.

Lifestyle interventions, as mentioned above, are typically the first-line treatment for GDM ^176, 177^. However, if these lifestyle changes are insufficient to maintain maternal glycemia at a safe level, medical treatments such as insulin therapy may be required ^178^. Additionally, lifestyle modifications or medications used to treat T2D have been successful in preventing or delaying the development of diabetes in women after GDM ^179^. However, in cases where insulin is not feasible or preferred, oral hypoglycaemic agents such as metformin or glyburide may be considered under close medical supervision ^9, 180^. Finally, monitoring and follow-up during pregnancy involve regular glucose monitoring, foetal surveillance, and multidisciplinary care coordination to adjust treatment as needed and mitigate complications. Recent studies have shown that telemedicine interventions have been found to effectively decrease glycaemic levels in patients with GDM and reduce the risk of complications, highlighting the need for close monitoring of the patients ^181, 182^.

## Further directions in research and conclusions

The rising incidence of GDM and its associated complications reflects the increasing prevalence of obesity among pregnant women. It could be debated that overweight women should strive for weight loss before conception. However, population-based surveys conducted in the UK have revealed that around half of pregnancies are unplanned ^183^. Similarly, a comparable proportion of women who actually planned their pregnancies fail to appropriately supplement, indicating that in many instances, adequate medical advice is neither sought nor provided during the pre-conceptional period ^184^. Furthermore, disparities in screening and diagnostic methodologies make it difficult to compare different studies between them. Therefore, the cost of the diagnosis of GDM can vary depending on the screening approach used, with some methods involving more expensive tests than others ^185^. Furthermore, debates persist regarding the appropriateness of subjecting pregnant women to such a substantial glucose challenge as the proposed in the 1 Step or 2 Steps approaches. An alternative could be the potential use of biomarkers that could offer a more cost-effective and less invasive means of diagnosis. However, research around this area is still insufficiently developed and it is not widely implemented in the routine clinical practice.

Following the confirmation of GDM, initial management typically involves lifestyle modifications. If these interventions prove ineffective, medical interventions may be considered as the next step. Unfortunately, evidence regarding interventions with lifestyle strategies for preventing GDM in pregnant women with and without risk factors is conflicting ^186^. This discrepancy likely stems from the considerable heterogeneity across trials in terms of cohort demographics and diagnostic criteria used to define the condition ^186^. Population-based studies investigating dietary or combined lifestyle measures have not consistently demonstrated improvements in GDM risk ^186– 188^. Similarly, trials involving physical activity strategies have yielded conflicting results, with some showing no significant impact on GDM incidence ^186^.

When lifestyle interventions fail to yield desired results, medical interventions may be deemed necessary. In cases where insulin administration is not feasible or preferred, the consideration of oral hypoglycemic agents such as metformin or glyburide may be considered, under meticulous medical supervision ^189, 190^. As for other medical interventions, Myoinositol supplementation has demonstrated potential in mitigating GDM risk, yet additional confirmatory studies are necessary ^191, 192^.

Beyond the management of GDM during pregnancy, recognizing the long-term health implications is crucial. Both mother and child face an elevated risk of developing type 2 diabetes (T2D), obesity, and metabolic syndrome (MetS) in the postnatal period. Thus, addressing the knowledge gaps regarding the metabolic pathways altered during GDM and the enduring metabolic imprint post-pregnancy is paramount. The identification of predictive biomarkers for T2D development in women with a history of GDM could guide preventive care. Moreover, advanced omics technologies, such as genomics, transcriptomics, and metabolomics, hold promise in elucidating the molecular signatures and biomarkers pivotal to the onset and progression of GDM. To improve maternal and offspring health outcomes and guide future research, several strategies should be prioritized. For example, research into novel biomarkers, which may offer a more precise and less invasive diagnostic alternative, should be encouraged. Given the inconsistency in lifestyle intervention outcomes, more tailored and culturally appropriate dietary and exercise interventions are needed. These interventions should be extended into the postpartum period to mitigate the elevated risk of T2D development, which is an aspect of GDM generally neglected by research. Postpartum glucose screening is essential but underutilized ^193^. Developing strategies to improve adherence to screening, such as digital health tools or structured follow-up programs, could reduce the long-term risk of T2DM ^194^. Longitudinal studies are also necessary to track the health outcomes of both mothers and their offspring, providing data that could inform future preventive measures. Psychological support for women with GDM is often overlooked, despite evidence suggesting that mental health integration into GDM management protocols can enhance both emotional well-being and glycemic control ^195^. Integrating mental health services within GDM management protocols, including counseling and stress management, could enhance both emotional well-being and glycemic control. Finally, educational campaigns directed at both healthcare providers and the public are critical to improving pre-conception health, increasing GDM screening, and promoting postpartum follow-up care ^196^. By integrating these strategies, we can address the rising incidence of GDM and improve both short- and long-term outcomes for mothers and their offspring.

## List of abbreviations

ADA - American Diabetes Association

AGE - Advanced Glycation End Product

ALA - Alpha-Linolenic Acid

AMPK - AMP-activated Protein Kinase

BMI - Body Mass Index

CVD - Cardiovascular Disease

GDM - Gestational Diabetes Mellitus

GCT - Glucose Challenge Test

GLUT4 – Glucose Transporter Type 4

FFAs - Free Fatty Acids

HAPO - Hyperglycemia and Adverse Pregnancy Outcomes

HbA1c - Hemoglobin A1c

HCY - Plasma homocysteine

hPL - Human Placental Lactogen

IADPSG - International Association of Diabetes and Pregnancy Study Groups

IDF - International Diabetes Federation

IL-6 - Interleukin-6

IRS1 - Insulin Receptor Substrate 1

LA - Linoleic Acid

LPS – Lipopolysaccharide

OGTT - Oral Glucose Tolerance Test

PAPP-A- Pregnancy-Associated Plasma protein-A

PCOS - Polycystic Ovary Syndrome

PI3K - Phosphatidylinositol 3-Kinase

PHAs - Polycyclic aromatic hydrocarbons

RDS - Respiratory Distress Syndrome

SNP - Single Nucleotide Polymorphism

TCF7L2 - Transcription Factor 7-like 2

TG_by_PG - Ratio of Triglycerides to Phosphoglycerides

T2DM - Type 2 Diabetes Mellitus

TNF-α - Tumor Necrosis Factor-alpha

UMFA - Unmetabolised folic acid

5,10-CH2-THF - 5, 10-methylene-tetrahydrofolate

5-CHO-THF - 5- formyltetrahydrofolate

5-MTHF - Methyltetrahydrofolate

## Data Availability

No data are associated with this article.
