# Supplementary material for: Gestational Diabetes Mellitus: Unveiling Maternal Health Dynamics from Pregnancy Through Postpartum Perspectives
Source: Open Res Eur. 2024 Nov 12;4:164. Originally published 2024 Aug 5. [Version 2] doi: 10.12688/openreseurope.18026.2 (PMC11443192; doi:10.12688/openreseurope.18026.2)
Supplement: Peer review history

# Reviewer report: Reviewer response for version 2

**DOI:** 10.21956/openreseurope.20373.r46147

**Published:** 2024-11-23

**Authors:** Xinhua Xiao

**Affiliations:** 1 Clinical Medicine, Endocrinologist, Peking Union Medical College, Chinese Academy of Medical Sciences, Beijing, China

The revised review by the author exhibits clearer logic, more accurate and comprehensive literature citations, and a deeper elaboration on the research background, current status, and significance.

Is the review written in accessible language?

Yes

Are all factual statements correct and adequately supported by citations?

Yes

Are the conclusions drawn appropriate in the context of the current research literature?

Yes

Is the topic of the review discussed comprehensively in the context of the current literature?

Partly

Reviewer Expertise:

Clinical Medicine, Endocrinologist

I confirm that I have read this submission and believe that I have an appropriate level of expertise to confirm that it is of an acceptable scientific standard.

# Reviewer report: Reviewer response for version 2

**DOI:** 10.21956/openreseurope.20373.r46145

**Published:** 2024-11-21

**Authors:** Jessica L Faulkner

**Affiliations:** 1 Augusta University, Augusta, USA

The authors have addressed the comments appropriately.

Is the review written in accessible language?

Yes

Are all factual statements correct and adequately supported by citations?

Partly

Are the conclusions drawn appropriate in the context of the current research literature?

Yes

Is the topic of the review discussed comprehensively in the context of the current literature?

Yes

Reviewer Expertise:

Preeclampsia and adverse outcomes of pregnancy

I confirm that I have read this submission and believe that I have an appropriate level of expertise to confirm that it is of an acceptable scientific standard.

# Reviewer report: Reviewer response for version 1

**DOI:** 10.21956/openreseurope.19481.r44756

**Published:** 2024-09-30

**Authors:** Dorota Darmochwał-Kolarz

**Affiliations:** 1 Clinical Provincial Hospital No. 2 for them St. Queen Jadwiga in Rzeszów, Institute of Medical Sciences, Medical College, University of Rzeszów, Rzeszów, Poland

The manuscript is well-written review study concerning risk factors and long-term consequences of gestational diabetes.

 The Authors describe some genetic variants and GDM risk, particularly a variant in the transcription factor 7-like 2 (TCF7L2) gene ^,^ MicroRNA-375 levels and single nucleotide polymorphisms (SNPs) in microRNA-375.

 There are some other genetic factors which are thought to be involved in the pathogenesis of gestational diabetes. More attention should be given and more detailed agents should be discussed in this section to describe the role of genetics in GDM.

Is the review written in accessible language?

Yes

Are all factual statements correct and adequately supported by citations?

Yes

Are the conclusions drawn appropriate in the context of the current research literature?

Yes

Is the topic of the review discussed comprehensively in the context of the current literature?

Yes

Reviewer Expertise:

Pre-eclampsia, Eclampsia

I confirm that I have read this submission and believe that I have an appropriate level of expertise to confirm that it is of an acceptable scientific standard.

# Response

**Published:** 2024-10-28

**Authors:** Marina Mora Ortiz

**Affiliations:** IMIBIC, Spain

**Reviewer 3. Dr. **Dorota Darmochwał-Kolarz **,**

**Reviewer 3:** The manuscript is a well-written review study concerning risk factors and long-term consequences of gestational diabetes. The Authors describe some genetic variants and GDM risk, particularly a variant in the transcription factor 7-like 2 (TCF7L2) gene ^,^ MicroRNA-375 levels and single nucleotide polymorphisms (SNPs) in microRNA-375. There are some other genetic factors which are thought to be involved in the pathogenesis of gestational diabetes. More attention should be given and more detailed agents should be discussed in this section to describe the role of genetics in GDM.  

**Authors:** Thank you very much for your thoughtful and constructive feedback. We appreciate your suggestion to expand on the genetic factors involved in the pathogenesis of gestational diabetes (GDM). Based on your comments, we have revised the manuscript to include a more comprehensive discussion of additional genetic agents associated with GDM risk. We hope that these additions enhance the depth and clarity of our discussion on genetic contributions to GDM.

# Reviewer report: Reviewer response for version 1

**DOI:** 10.21956/openreseurope.19481.r43325

**Published:** 2024-09-30

**Authors:** Dorota Darmochwał-Kolarz

**Affiliations:** 1 Clinical Provincial Hospital No. 2 for them St. Queen Jadwiga in Rzeszów, Institute of Medical Sciences, Medical College, University of Rzeszów, Rzeszów, Poland

The manuscript is well-written review study concerning risk factors and long-term consequences of gestational diabetes.

 The Authors describe some genetic variants and GDM risk, particularly a variant in the transcription factor 7-like 2 (TCF7L2) gene ^,^ MicroRNA-375 levels and single nucleotide polymorphisms (SNPs) in microRNA-375.

 There are some other genetic factors which are thought to be involved in the pathogenesis of gestational diabetes. More attention should be given and more detailed agents should be discussed in this section to describe the role of genetics in GDM.

Is the review written in accessible language?

Yes

Are all factual statements correct and adequately supported by citations?

Yes

Are the conclusions drawn appropriate in the context of the current research literature?

Yes

Is the topic of the review discussed comprehensively in the context of the current literature?

Yes

Reviewer Expertise:

Pre-eclampsia, Eclampsia

I confirm that I have read this submission and believe that I have an appropriate level of expertise to confirm that it is of an acceptable scientific standard.

# Reviewer report: Reviewer response for version 1

**DOI:** 10.21956/openreseurope.19481.r43688

**Published:** 2024-09-14

**Authors:** Xinhua Xiao

**Affiliations:** 1 Clinical Medicine, Endocrinologist, Peking Union Medical College, Chinese Academy of Medical Sciences, Beijing, China

This article focused on gestational diabetes mellitus (GDM), meticulously outlining its prevalence, diagnosis, Pathophysiology,and the profound health implications it poses for both mothers and their offspring. While the title of the article is mainly about the postpartum health dynamics experienced by pregnant women, the core content predominantly revolves around the prevalence, diagnostic methodologies, therapeutic interventions, and biomarkers associated with GDM during gestation. Regrettably, the discourse on the postpartum period,is somewhat described, primarily focusing on the lingering effects of GDM on maternal and offspring health over the long term. This limitation underscores the need for a more comprehensive exploration of the immediate and intermediate postpartum experiences, including any potential changes in glycemic control, lifestyle adjustments, and the emotional and psychological implications of transitioning from pregnancy to the postnatal stage amidst the backdrop of GDM. Consider changing the title or adjusting the content of the article.

 The abstract of the review mentions its aim to foster further investigations in the field of GDM and to improve the health outcomes for mothers and their offspring. However, the article notably lacks a significant focus on outlining specific strategies or approaches for achieving such improvements.

 Furthermore, the cited references within the review exhibit a tendency towards utilizing sources from an earlier period, with a lesser representation of recent publications. Ideally, as a comprehensive review, it is expected that at least 80% of the cited references should encompass articles published within the last five years to ensure the timeliness and relevance of the information presented. Please incorporate more recent studies, which would not only reflect the latest advancements and discoveries in GDM research but also strengthen the foundation for developing effective interventions.

 Part 2: “A comprehensive exploration of the risk factors shaping susceptibility to GDM”. The risk factors affecting susceptibility to GDM is not comprehensive enough. The article only describes common clinical indicators s, but some biochemical indicators such as FPG, HBA1c, LDL-C, etc., are not included in the discussion. And certain risk factors such as metabolites that can predict the risk of GDM could also be included. See Yeyi Zhu, et al.Diabetes 2022; 71:1807-18179(ref 1)

 Part 4: “Short-term and long-term complications derived from GDM in the mothers and children”，In this section, the first and second paragraphs, which do not correspond to the subtitle, discuss whether GDM and preeclampsia are independently related or have common risk factors. It is recommended to clarify the short-term effects for mother and child and the long-term effects for mother and child respectively.

 Part 5: “Biomarker signatures in GDM”. This part mainly describes some markers of GDM, but it is not complete. It is suggested to divide it into sample elaboration or detection method elaboration. In terms of samples, it can be divided into what markers are present in the mother's blood and what markers are present in the placenta or umbilical cord blood. In terms of detection methods, it can be divided into genomics, metabolomics and proteomics. Or deleting this section, because the markers described in the article only describe correlations, not to the extent that they are relevant to diagnosis, prediction, and prognosis, and are not relevant to the topic of the article.

 Co-reviewer comments

 This paper presents a clear logical framework and in-depth analysis when discussing its topic. However, when I look at the article as a whole, I find that the actual discussion content of the article is somewhat different from the focus indicated by the title. To ensure that readers can accurately anticipate and understand the core content of the article, it is recommended that authors review and adjust the title to more accurately reflect the main idea of the article. With this change, the readability and academic rigor of the article can be significantly improved.

 About the timeliness of literature citations:

 This paper has done some work in citing the relevant literature, which undoubtedly provides a solid theoretical basis for the discussion of this paper. However, it is worth noting that some of the references are older and fail to fully reflect the latest research results and developments in this field in the past five years. In view of the fact that review articles are usually required to cover the latest and most cutting-edge academic progress, it is recommended that authors increase the literature citations in the last five years, and appropriately reduce or adjust some old literature citations, so as to ensure that the articles can keep up with the academic frontier and present readers with a more comprehensive and novel research perspective.

 About the improvement of the chart and the expansion of the table content:

 The charts in this paper are beautifully designed and the data are clearly presented, which effectively enhances the persuasiveness and readability of the article. In particular, the design of the chart section deserves praise. However, with regard to Table 2, I think there is room for further expansion. Authors are advised to consider adding more relevant data items or classifications to the table in order to present the findings and comparative analysis more fully. This change will help to improve the information and usefulness of the table and further enhance the overall quality of the article.

Is the review written in accessible language?

Yes

Are all factual statements correct and adequately supported by citations?

Yes

Are the conclusions drawn appropriate in the context of the current research literature?

Yes

Is the topic of the review discussed comprehensively in the context of the current literature?

Partly

Reviewer Expertise:

Clinical Medicine, Endocrinologist

I confirm that I have read this submission and believe that I have an appropriate level of expertise to confirm that it is of an acceptable scientific standard, however I have significant reservations, as outlined above.

## References

[1] : Prevalence of Gestational Diabetes and Risk of Progression to Type 2 Diabetes: a Global Perspective. *Curr Diab Rep* .2016;16(1) : 10.1007/s11892-015-0699-x 7 10.1007/s11892-015-0699-x 26742932 PMC6675405

# Response

**Published:** 2024-10-28

**Authors:** Marina Mora Ortiz

**Affiliations:** IMIBIC, Spain

**Reviewer 2. Dr. Xinhua Xiao** **Reviewer 2: **This article focused on gestational diabetes mellitus (GDM), meticulously outlining its prevalence, diagnosis, Pathophysiology, and the profound health implications it poses for both mothers and their offspring. While the title of the article is mainly about the postpartum health dynamics experienced by pregnant women, the core content predominantly revolves around the prevalence, diagnostic methodologies, therapeutic interventions, and biomarkers associated with GDM during gestation. Regrettably, the discourse on the postpartum period, is somewhat described, primarily focusing on the lingering effects of GDM on maternal and offspring health over the long term. This limitation underscores the need for a more comprehensive exploration of the immediate and intermediate postpartum experiences, including any potential changes in glycemic control, lifestyle adjustments, and the emotional and psychological implications of transitioning from pregnancy to the postnatal stage amidst the backdrop of GDM. Consider changing the title or adjusting the content of the article.

**Authors response:** Thank you very much for the feedback. We have revised the content following the advice provided by the reviewer. Specifically, we have expanded the section related to postpartum GDM by including a more comprehensive exploration of the immediate and intermediate postpartum experiences. This includes a detailed discussion of the psychological and emotional challenges faced by women transitioning from pregnancy to the postnatal period, with particular emphasis on mental health, as suggested. Additionally, we have incorporated new content on the benefits of breastfeeding in mitigating the long-term risk of developing type 2 diabetes (T2DM), providing evidence of its protective role in the postpartum period. These revisions can be found in the section titled *“Short-term and Long-term Complications Derived from GDM in Mothers and Children.”* We believe these updates address the reviewer's concerns and enrich the manuscript by providing a more balanced discussion of both the gestational and postpartum dynamics of GDM.  

**Reviewer 2: **The abstract of the review mentions its aim to foster further investigations in the field of GDM and to improve the health outcomes for mothers and their offspring. However, the article notably lacks a significant focus on outlining specific strategies or approaches for achieving such improvements.

**Authors response:** We appreciate your comments. In response to your observation that the manuscript "lacks a significant focus on outlining specific strategies or approaches for improving health outcomes for mothers and their offspring," we have taken steps to address this issue comprehensively. In the revised manuscript, we have now integrated specific strategies for improving maternal and offspring health outcomes in the context of GDM. These strategies are detailed in the "Further Directions in Research and Conclusions" section. This is the new section: “Beyond the management of GDM during pregnancy, recognizing the long-term health implications is crucial. Both mother and child face an elevated risk of developing type 2 diabetes (T2D), obesity, and metabolic syndrome (MetS) in the postnatal period. Thus, addressing the knowledge gaps regarding the metabolic pathways altered during GDM and the enduring metabolic imprint post-pregnancy is paramount. The identification of predictive biomarkers for T2D development in women with a history of GDM could guide preventive care. Moreover, advanced omics technologies, such as genomics, transcriptomics, and metabolomics, hold promise in elucidating the molecular signatures and biomarkers pivotal to the onset and progression of GDM. To improve maternal and offspring health outcomes and guide future research, several strategies should be prioritized. For example, research into novel biomarkers, which may offer a more precise and less invasive diagnostic alternative, should be encouraged. Given the inconsistency in lifestyle intervention outcomes, more tailored and culturally appropriate dietary and exercise interventions are needed. These interventions should be extended into the postpartum period to mitigate the elevated risk of T2D development, which is an aspect of GDM generally neglected by research. Postpartum glucose screening is essential but underutilized[IF1]. Developing strategies to improve adherence to screening, such as digital health tools or structured follow-up programs, could reduce the long-term risk of T2D[IF2] . Longitudinal studies are also necessary to track the health outcomes of both mothers and their offspring, providing data that could inform future preventive measures. Psychological support for women with GDM is often overlooked, despite evidence suggesting that mental health integration into GDM management protocols can enhance both emotional well-being and glycemic control.[IF3]  Integrating mental health services within GDM management protocols, including counselling and stress management, could enhance both emotional well-being and glycemic control. Finally, educational campaigns directed at both healthcare providers and the public are critical to improving pre-conception health, increasing GDM screening, and promoting postpartum follow-up care[IF4]. By integrating these strategies, we can address the rising incidence of GDM and improve both short- and long-term outcomes for mothers and their offspring.” These additions provide a clearer and more focused roadmap for future research and practical interventions aimed at improving health outcomes for mothers and their offspring. We hope that these revisions align with your suggestions for a more comprehensive focus on strategies to address the challenges posed by GDM. Thank you once again for your insightful comments, which have greatly helped in improving the clarity and focus of the manuscript.  

**Reviewer 2: **Furthermore, the cited references within the review exhibit a tendency towards utilizing sources from an earlier period, with a lesser representation of recent publications. Ideally, as a comprehensive review, it is expected that at least 80% of the cited references should encompass articles published within the last five years to ensure the timeliness and relevance of the information presented. Please incorporate more recent studies, which would not only reflect the latest advancements and discoveries in GDM research but also strengthen the foundation for developing effective interventions.

**Authors response: **We appreciate your suggestion The older citations are included because they are widely accepted references by the scientific community regarding GDM. However, we have expanded the number of citations and included numerous articles published within the last 5 years (i.e., Table 4).  

**Reviewer 2: **Part 2: “A comprehensive exploration of the risk factors shaping susceptibility to GDM”. The risk factors affecting susceptibility to GDM is not comprehensive enough. The article only describes common clinical indicators s, but some biochemical indicators such as FPG, HBA1c, LDL-C, etc., are not included in the discussion. And certain risk factors such as metabolites that can predict the risk of GDM could also be included. See Yeyi Zhu, et al.Diabetes 2022; 71:1807-18179(ref 1)  

**Authors response: **We appreciate your insightful feedback regarding the exploration of risk factors shaping susceptibility to gestational diabetes mellitus (GDM) in our manuscript. We acknowledge the importance of providing a comprehensive overview of both risk factors and biomarkers associated with GDM. In our revised section, we aim to clarify the distinction between risk factors and biomarkers, as these terms are often mistakenly used interchangeably. The terms "risk factors" and "biomarkers" are often used interchangeably in discussions surrounding gestational diabetes mellitus (GDM), but they represent different concepts in the assessment of disease susceptibility. Risk factors for GDM are characteristics or conditions that are statistically associated with an increased likelihood of developing the condition. These include demographic factors such as advanced maternal age, elevated body mass index (BMI), and a family history of diabetes, as well as lifestyle factors like diet and physical activity levels. While these factors can indicate a higher susceptibility to GDM, they do not provide specific measurements or indicators of the physiological state of an individual. In contrast, biomarkers are measurable indicators of biological processes or conditions that can predict or reflect the likelihood of developing GDM. For instance, fasting plasma glucose (FPG), haemoglobin A1c (HbA1c), and specific lipid profiles can serve as biomarkers for GDM risk, as they provide quantifiable data on an individual's metabolic state. These biomarkers can enhance the predictive capability for GDM by offering a more direct assessment of the physiological changes that occur during pregnancy. While risk factors highlight the demographic and lifestyle characteristics that may predispose individuals to GDM, biomarkers provide a more precise means of assessing and predicting the condition. Future research should aim to integrate both risk factors and biomarkers to improve the early identification and management of GDM in pregnant women. We will expand our discussion to include recent findings from the literature, such as those presented by Yeyi Zhu et al. (2022), which highlight metabolites that can predict GDM development. By integrating this information in the biomarkers section, we will provide a more thorough examination of both risk factors and biomarkers associated with GDM. Thank you once again for your valuable insights, which have guided us in refining our discussion on the risk factors and biomarkers for GDM.  

**Reviewer: **Part 4: “Short-term and long-term complications derived from GDM in the mothers and children”，In this section, the first and second paragraphs, which do not correspond to the subtitle, discuss whether GDM and preeclampsia are independently related or have common risk factors. It is recommended to clarify the short-term effects for mother and child and the long-term effects for mother and child respectively.

**Authors response: **thank you for your suggestion. In the new manuscript, we have included a paragraph detailing the short and long complications of GDM.  

**Reviewer 2: **Part 5: “Biomarker signatures in GDM”. This part mainly describes some markers of GDM, but it is not complete. It is suggested to divide it into sample elaboration or detection method elaboration. In terms of samples, it can be divided into what markers are present in the mother's blood and what markers are present in the placenta or umbilical cord blood. In terms of detection methods, it can be divided into genomics, metabolomics and proteomics. Or deleting this section, because the markers described in the article only describe correlations, not to the extent that they are relevant to diagnosis, prediction, and prognosis, and are not relevant to the topic of the article.

**Authors response: **This section has been rephrased and expanded, and the information has been reflected as Reviewer 2 proposed. See Table 4.

# Reviewer report: Reviewer response for version 1

**DOI:** 10.21956/openreseurope.19481.r43319

**Published:** 2024-09-12

**Authors:** Jessica L Faulkner

**Affiliations:** 1 Augusta University, Augusta, USA

The current manuscript provides an overview of GDM risk factors and potential mechanisms at play using recent references and up to date information. There are some minor comments below to improve the review.

1. The Figure 3 is a bit confusing. The dysfunction is not clearly separated from what are normal physiological processes. The figure should depict the normal physiological process of glucose synthesis, demand and transfer and then candidate mechanisms whereby the fetal placental unit disrupts this process and causes spontaneous insulin resistance
2. “The placental transfer system does, in fact, allow only around 3% of the mater’s fatty acids to reach the developing foetus; this is far less efficient than for glucose”. This sentence has a typo.
3. Please don’t use the term “fatter”, higher adipose content/mass etc would be more appropriate
4. The association of leptin with GDM is a bit more complicated than is listed in this manuscript. The authors are referred to Elgazzaz et al. AJP Cell. 2024 ”Implications of pregnancy on cardiometabolic disease risk: preeclampsia and gestational diabetes”

Is the review written in accessible language?

Yes

Are all factual statements correct and adequately supported by citations?

Partly

Are the conclusions drawn appropriate in the context of the current research literature?

Yes

Is the topic of the review discussed comprehensively in the context of the current literature?

Yes

Reviewer Expertise:

Preeclampsia and adverse outcomes of pregnancy

I confirm that I have read this submission and believe that I have an appropriate level of expertise to confirm that it is of an acceptable scientific standard, however I have significant reservations, as outlined above.

# Response

**Published:** 2024-10-28

**Authors:** Marina Mora Ortiz

**Affiliations:** IMIBIC, Spain

**Reviewer 1 Dr. Jessica L Faulkner**   The current manuscript provides an overview of GDM risk factors and potential mechanisms at play using recent references and up to date information. There are some minor comments below to improve the review. **Reviewer: 1.**     The Figure 3 is a bit confusing. The dysfunction is not clearly separated from what are normal physiological processes. The figure should depict the normal physiological process of glucose synthesis, demand and transfer and then candidate mechanisms whereby the fetal placental unit disrupts this process and causes spontaneous insulin resistance **Authors: **Dear Dr. Faulkner, Thank you for your valuable feedback on our manuscript. We greatly appreciate your insightful comments regarding this topic. Upon reviewing your suggestion, we have decided to include a new table (now, Table 3) that compares the normal physiological processes during pregnancy with the alterations seen in GDM. This table is intended to delineate the differences between normal pregnancy and GDM, as you recommended. We have chosen to retain Figure 3 in its original form, as it focuses specifically on the processes occurring in GDM. We feel that this figure plays an important role in visually illustrating the mechanisms involved in GDM, while the new table serves to complement it by clearly comparing normal physiology with the dysfunctions seen in GDM. In addition, we have further clarified in the text what occurs during normal pregnancy and the changes observed during GDM. These are the key points addressed in the text: 

1. **Increased Glucose Production**: We acknowledge the necessity for mothers to enhance glucose production by approximately 30% by the end of gestation, primarily through hepatic gluconeogenesis. This adaptation is crucial for meeting the heightened fasting energy demands during pregnancy, ensuring a continuous glucose supply to both maternal tissues and the developing fetus.
2. **Maternal Plasma Glucose Levels**: We would like to clarify that despite the increase in glucose production, maternal plasma glucose concentrations are generally lower due to factors such as expanded plasma volume and increased glucose utilization by the fetoplacental unit in late gestation. This phenomenon highlights the complex regulatory mechanisms at play during pregnancy.
3. **Insulin Sensitivity and Secretion**: In healthy pregnancies, we observe a reduction in insulin sensitivity by approximately 50% by the end of gestation. However, this is effectively compensated by a 2- to 3-fold increase in insulin secretion, which helps maintain euglycemia. We believe this balance is a critical aspect of normal metabolic adaptation during pregnancy.
4. **Pathophysiology of GDM**: Regarding gestational diabetes, we agree that its pathophysiology is characterized by heightened insulin resistance and impaired β-cell function. Evidence suggests that β-cell defects may precede conception, becoming evident under the metabolic stress of pregnancy. This will also be highlighted in the figure.
5. **Endogenous Glucose Production in Normal and GDM Pregnancies**: We emphasize the importance of the hyperinsulinaemic-euglycaemic clamp studies. In women with normal glucose tolerance, endogenous glucose production is nearly completely suppressed in response to insulin infusion, indicating effective glucose regulation. In contrast, women who develop GDM show significantly less suppression of endogenous glucose production (approximately 80-85%), which contributes to postprandial hyperglycemia. This differential response underscores the metabolic challenges faced by women with GDM.

We hope this clarifies our work and adequately addresses your concerns. Thank you for your thoughtful review, and we look forward to your further feedback. **Reviewer 1:**      “The placental transfer system does, in fact, allow only around 3% of the mater’s fatty acids to reach the developing foetus; this is far less efficient than for glucose”. This sentence has a typo. **Authors:**

 Thank you very much for pointing out the typo in the sentence. We have corrected it, and the revised sentence now reads: *"The placental transfer system does, in fact, allow only around 3% of the mother's fatty acids to reach the developing foetus; this is far less efficient than for glucose."* We appreciate your attention to detail. **Reviewer 1: **Please don’t use the term “fatter”, higher adipose content/mass etc would be more appropriate **Authors:**

 Thank you for your suggestion regarding the terminology. We have replaced "fatter" with more precise language. The revised sentence now reads: *"Higher adipose mass and/or larger newborns, as well as those with greater cord serum C-peptide levels, carry a higher risk."* We agree that "higher adipose mass" is a more appropriate and accurate term, and we appreciate your feedback. **Reviewer 1: **The association of leptin with GDM is a bit more complicated than is listed in this manuscript. The authors are referred to Elgazzaz et al. AJP Cell. 2024 ”Implications of pregnancy on cardiometabolic disease risk: preeclampsia and gestational diabetes” **Authors:** Thank you for your valuable feedback regarding the association of leptin with gestational diabetes mellitus (GDM). We appreciate your reference to your work “Elgazzaz et al. (2024)” and acknowledge that the role of leptin in GDM is indeed complex. In the revised manuscript, we have expanded our discussion on this topic to reflect the intricacies involved. Specifically, we address the following key points:

1. Leptin Functionality
2. Leptin Levels in GDM
3. Influence of Insulin Resistance and Fatty Acids
4. Timing of GDM Onset
5. Fetal Leptin Production

We hope these additions provide a more comprehensive perspective on the relationship between leptin and GDM, reflecting the complexities highlighted in your review. Thank you again for your insightful comments, which have helped enhance the clarity and depth of our manuscript.
